# A general framework for genome rearrangement with biological constraints

**DOI:** 10.1186/s13015-019-0149-4

**Published:** 2019-07-19

**Authors:** Pijus Simonaitis, Annie Chateau, Krister M. Swenson

**Affiliations:** 1 CNRS, LIRMM, Université Montpellier, 161 Rue Ada, 34392 Montpellier, France; 2 Institut de Biologie Computationnelle (IBC), Montpellier, France

**Keywords:** Double cut and join (DCJ), Weighted genome rearrangement, Breakpoint graph, Graph edit distance, Edge switch

## Abstract

This paper generalizes previous studies on genome rearrangement under biological constraints, using double cut and join (DCJ). We propose a model for weighted DCJ, along with a family of optimization problems called $\varphi$-MCPS (Minimum Cost Parsimonious Scenario), that are based on labeled graphs. We show how to compute solutions to general instances of $\varphi$-MCPS, given an algorithm to compute $\varphi$-MCPS on a circular genome with exactly one occurrence of each gene. These general instances can have an arbitrary number of circular and linear chromosomes, and arbitrary gene content. The practicality of the framework is displayed by presenting polynomial-time algorithms that generalize the results of Bulteau, Fertin, and Tannier on the Sorting by wDCJs and indels in intergenes problem, and that generalize previous results on the Minimum Local Parsimonious Scenario problem.

## Introduction

### Context

The practical study of genome rearrangement scenarios has been limited by a lack of mathematical models capable of incorporating biological constraints, since foundational models focused on minimum length scenarios transforming one genome into another. In the modern age, where the collection of fully assembled and annotated genomes is ever-increasing, there is the need for the development of more elaborate mathematical models that consider the data from multiple biological experiments.

One way to incorporate biological information into the inference of evolutionary scenarios is to consider models that weight rearrangements according to their likelihood of occurring; a breakpoint may be more likely to occur in some intergenic regions than others. To this end, the study of length-weighted reversals was started in the late nineties by Blanchette et al. [1]. Baudet et al. present a summary of work done in this area, along with work on reversals centered around the origin of replication [2]. Recently, Tannier has published a series of papers focused on weighting intergenic regions by their length in nucleotides. In [3], Biller et al. pointed out that, according to the Nadeau–Taylor model of uniform random breakage [4, 5], a breakpoint is more likely to occur in a longer intergenic region. Subsequent papers by Fertin et al. [6], and Bulteau et al. [7] present algorithmic results for models that take into account the length of intergenic regions. Using Hi-C data [8], Veron et al. along with our own study, have pointed out the importance of weighting pairs of breakpoints according to how close they tend to be in physical space [9, 10]. In order to use this physical constraint, we partitioned intergenic regions into co-localized areas, and developed algorithms for computing distances that minimize the number of rearrangements that operate on breakpoints between different areas [11, 12].

Much of this work is based on the mathematically clean model for genome rearrangement called *Double Cut and Join*, or *DCJ* [13, 14]. Genomes are partitioned into *n* orthologous syntenic blocks that we will simply call *genes*. Each gene is represented by two extremities, and each chromosome is represented by an ordering of these extremities. Those extremities that are adjacent in this ordering are paired, and transformations of these pairs occur by swapping extremities of two pairs. DCJ can naturally be interpreted as a graph edit model with the use of the *breakpoint graph*, where there is an edge between gene extremities *a* and *b* for each adjacent pair. A DCJ operation replaces an edge pair $\big \{\{a,b\}, \{c,d\}\big \}$ of the graph by $\big \{\{a,c\}, \{b,d\}\big \}$ or $\big \{\{a,d\}, \{b,c\}\big \}$. This edge edit operation on a graph is called a *2-break*.

This paper establishes a general framework for weighting rearrangements. The results are based on the problem of transforming one labeled graph into another through a scenario of operations, each weighted by an arbitrary function $\varphi$. The problem, called $\varphi$ -Minimum Cost Parsimonious Scenario (or $\varphi$-MCPS), asks for a scenario with a minimum number of 2-breaks, such that the sum of the costs for the operations is minimized.

### Applications of our framework

While our framework is general, we use it to render two previous studies more practical. The first study is our work relating the likelihood of rearrangement breakpoints to the physical proximity in the nucleus [11]. This work is based on the hypothesis that two breakpoints could be confused when they are physically close. The model in this study labels the breakpoint graph edges (corresponding to intergenic regions) with fixed “colors”, and the cost of a DCJ has a weight of one if the labels are different and a weight of zero if they are the same. Using that cost function, we colored intergenic regions by grouping them according to their physical proximity, as inferred by Hi-C data. Although this technique of grouping proved to make biological sense [10, 12], it is far from ideal since much of the information given by the Hi-C data is lost in the labeling, and it is not immediately clear how to best compute the grouping. Our results here bypass the complexity of grouping by allowing each DCJ to be weighted by the values taken directly from the Hi-C contact maps. We give an algorithm for $\varphi$-MCPS on a breakpoint graph with an arbitrary $\varphi$ and fixed edge labels, that runs in $O(n^5)$ time in the worst case but has better parameterized complexity in practice (see Example 1). We give in “Practical matters” section other reasons why the running times for this algorithm should remain practical.

The second study that we improve is that of Bulteau et al. [7]. Their biological constraint is based on the number of nucleotides in the intergenic regions containing breakpoints; they compute parsimonious scenarios that minimize the number of nucleotides inserted and deleted in intergenic regions. Their algorithm is restricted to instances where the breakpoint graph has only cycles (and no paths—sometimes referred to as *co-tailed* genomes). Using their $O(n \log n)$ algorithm, our framework gives an $O(n^3)$ algorithm on any breakpoint graph (see Example 3).

This is an example of how our framework simplifies algorithm design on weighted DCJs. For a weight function adhering to our general criteria of “Cost-constrained 2-breaks” section, future algorithm designers now need only to concentrate on developing an efficient algorithm that works on a single cycle of a breakpoint graph. Thanks to Theorem 3, they will get a polynomial time algorithm that works on a general instance for free. “*α*-approximation for *φ*-MCPS” section shows that the same is true for approximation algorithms.

**Fig. 1:**
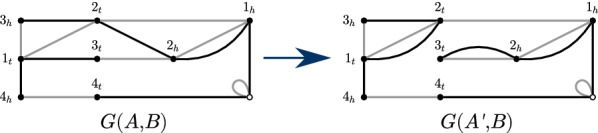
Eulerian 2-edge-color multi-graphs for genomes $A = \big (\{3_{t},1_{t}\},\{1_{h},2_{h}\},\{2_{t},3_{h}\}\big )$, $\big (\{4_{t}\},\{4_{h},1_{t}\},\{1_{h}\}\big )$, $B=\big (\{1_{h},2_{h}\},\{2_{t},1_{t}\}\big )$, $\big (\{3_{t},2_{h}\},\{2_{t},1_{h}\},\{1_{t},3_{h}\}\big )$, and $A'=\big (\{3_{t},2_{h}\},\{2_{t},1_{t}\},\{1_{h},2_{h}\},\{2_{t},3_{h}\}\big )$, $\big (\{4_{t}\},\{4_{h},1_{t}\},\{1_{h}\}\big )$. Edges adjacent to a special vertex $\circ$ represent the endpoints of linear chromosomes (e.g. black edges $\{1_{h},\circ \}$ and $\{4_t,\circ \}$). Extra edges are added for the missing genes (e.g. the black edge $\{2_{t},2_{h}\}$ and the gray edge $\{4_h,4_t\}$), called *ghost adjacencies* in [15]. In the genomes *A* and $A'$, gene 1 is repeated twice, and the operation transforming *A* into $A'$ is an insertion of a gene 2, corresponding to the 2-break $G(A,B)\rightarrow G(A',B)$. A DCJ scenario transforming $A'$ into the linear genome *B* includes a deletion of a gene 4

This paper is based on general results we obtain on weighted transformations of edge-labeled multi-graphs. The permitted transformations can change the connectivity of the graph through a 2-break, or change the edge labels, or both. This model not only proves to be powerful enough to subsume the previously mentioned results, but also offers other advantages. It is flexible enough so that DCJ costs can be based on the labels of edges in the breakpoint graph, or on the labels of the vertices, or a combination of both. Also, since single-gene insertions and deletions can be represented as “ghost” adjacencies [15], all of this paper applies to genomes where genes could be missing in one genome or the other. Most results can be applied to genomes with duplicate genes (as depicted in Fig. 1).

### Our model and general results

The foundation of this paper is a model for cost-constraining scenarios of degree preserving graph transformations, called 2-breaks, that are also known as edge swaps, switches, rewirings, or flips [16]. A 2-break transforms a graph by replacing two edges $\{u,v\}$ and $\{q,s\}$ by $\{u,q\}$ and $\{v,s\}$. These transformations have been studied not only in a restricted setting for genome rearrangement [14, 17] and sorting strings by mathematical transpositions [18, 19], but also in the more general settings of generating random networks [16] and network design [20, 21].

**Fig. 2:**
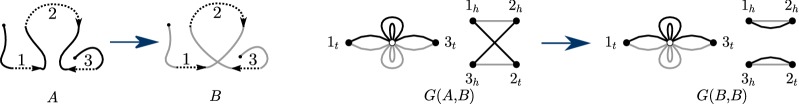
Two genomes with their respective sets of adjacencies $\big \{\{1_t\}, \{1_h, 2_t\}, \{2_h,3_h\}, \{3_t\}\big \}$ and $\big \{\{1_t\}, \{1_h, 2_h\}, \{2_t,3_h\}, \{3_t\}\big \}$. A DCJ $\{1_{h},2_{t}\},\{2_{h},3_{h}\}\rightarrow \{1_{h},2_{h}\},\{2_{t},3_{h}\}$ transforms *A* into *B*. The transformation $G(A,B)\rightarrow G(B,B)$ is a 2-break and *G*(*B*, *B*) is a terminal graph

Our results are about the transformation of an arbitrary multi-graph *G* into another one *H* having the same degree sequence. We find it convenient to reason in a setting, where we are given an Eulerian 2-edge-colored multi-graph with black and gray edges, the black edges being from *G* and the gray from *H*. We transform the connectivity of the black edges into the connectivity of the gray edges using a sequence of 2-breaks. Therefore, whenever we use the word *graph*, *path* (respectively *cycle*), we are referring to an Eulerian 2-edge-colored multi-graph, a path (respectively cycle) that alternates between black and gray edges. Naturally, a *cycle decomposition* of a graph is a partition of the edges of an Eulerian 2-edge-colored multi-graph into a set of alternating cycles. A *breakpoint graph* is a graph with a vertex for each gene extremity—each incident to exactly one gray and one black edge—along with one chromosome endpoint vertex $\circ$ that could have degree as high as 2*n* (see Fig. 2). “DCJ scenarios for genomes and breakpoint graphs” section introduces the breakpoint graph in detail, and defines the Double Cut and Join (DCJ) model.

Our model for weighting 2-breaks is primarily based on a graph labeling, a set $\mathcal {O}$ of valid operations, and a weight function $\varphi : \mathcal {O} \rightarrow \mathbb {R}_+$. Roughly speaking, a labeled input graph can be transformed through a series of operations in $\mathcal {O}$, where an operation can change the connectivity of the black edges of the graph, and/or change the labels of the edges. Any weight function $\varphi$ defines an optimization problem $\varphi$-MCPS, which asks for a scenario that minimizes the total weight of the operations. This model subsumes many previously studied weighted DCJ models, as described in “Examples of the cost-constrained DCJ problems in the literature” section.

The spine of our results is built from successive theorems that speak to the decomposability into subproblems of a $\varphi$-MCPS instance. Lemma 3 shows that a parsimonious scenario of 2-breaks transforming the black edges into the gray implies a Maximum Alternating Edge-disjoint Cycle Decomposition (or MAECD) [22]. Theorem 1 says that an optimal solution to $\varphi$-MCPS can be found using solutions to the MAECD problem, so that if $\varphi$-MCPS can be solved on a simple alternating cycle, then it can be solved on any instance. Theorem 2 says that an optimal solution to $\varphi$-MCPS on a simple alternating cycle can be found using a solution to the $\varphi$-MCPS problem on what we call a *circle*, that is, an alternating cycle that does not visit the same vertex twice (see Fig. 4).

Under the common genome model, where each gene occurs exactly once in each genome, a relationship exists between parsimonious DCJ scenarios and solutions to MAECD on a breakpoint graph [14, 23]. We exploit this link in “[Sec Sec13]” section. Theorem 3 ties everything together; an amortized analysis shows that, given an $O(r^t)$ algorithm for computing $\varphi$-MCPS on a circle with *r* edges, $\varphi$-MCPS can be calculated on a breakpoint graph in $O(n^{t+1})$ time.

Under a more general genome model, that allows for changes in copy numbers of genes (e.g. insertions, deletions, and duplications), the spine of our results still holds due to the convenient representation of missing genes as *ghost adjacencies* in an Eulerian 2-edge-colored multi-graph [15] (see Fig. 1). All of our results hold for pairs of genomes with non-duplicated genes, but unequal gene content. Indeed, a breakpoint graph (i.e. graph with limited degree for most nodes) can still represent the pair of genomes in this case.

Caprara proved that MAECD is NP-Hard for Eulerian 2-edge-colored multi-graphs where each vertex is incident to at most two gray and two black edges (which is the case when there are two copies of each gene) [22]. We present a simple integer linear program (or ILP) that solves $\varphi$-MCPS for these types of graphs, given a method to solve $\varphi$-MCPS on a circle. This ILP is likely to be unwieldy in general, since the number of variables is exponential in the number of simple alternating cycles. In the case of breakpoint graphs on specific genomes, this may not always be intractable, as the number of duplicate genes may be limited. See “Practical matters” section for a discussion of these practical matters.

## DCJ scenarios for genomes and breakpoint graphs

A *genome* consists of *chromosomes* that are linear or circular orders of genes separated by potential *breakpoint* regions. In Fig. 2 the tail of an arrow represents the *tail extremity*, and the head of an arrow represents the *head extremity* of a gene. We can represent a genome by a set of *adjacencies* between the gene extremities. An *adjacency* is either *internal*: an unordered pair of the extremities that are adjacent on a chromosome, or *external*: a single extremity adjacent to one of the two ends of a linear chromosome. In what follows we will suppose that two genomes *A* and *B* are partitioned into *n* genes each occurring exactly once in each genome, and our goal will be to transform *A* into *B* using a sequence of DCJs.

### **Definition 1**

(*double cut and join*) A DCJ cuts one or two breakpoint regions and joins the resulting ends of the chromosomes back in one of the four following ways: $\{a,b\},\{c,d\}\rightarrow \{a,c\},\{b,d\}$; $\{a,b\},\{c\}\rightarrow \{a,c\},\{b\}$; $\{a,b\}\rightarrow \{a\},\{b\}$; and $\{a\},\{b\}\rightarrow \{a,b\}$.

We represent the pairs of the genomes with a help of a breakpoint graph [13, 17].

### **Definition 2**

(*breakpoint graph*) *G*(*A*, *B*) for genomes *A* and *B* is a 2-edge-colored Eulerian undirected multi-graph. *V* consists of 2*n* gene extremities and an additional vertex $\circ$. For every internal adjacency $\{a,b\}\in A$ (respectively $\{a,b\}\in B$) there is a black (respectively gray) edge $\{a,b\}$ in *G*(*A*, *B*) and for every external adjacency $\{a\}\in A$ (respectively $\{a\}\in B$) there is a black (respectively gray) edge $\{a,\circ \}$ in *G*(*A*, *B*). There is a number of black and gray loops $\{\circ , \circ \}$ ensuring that $d^{b}(G(A,B),\circ )=d^{g}(G(A,B),\circ )=2n$.

## 2-Break scenarios for 2-edge-colored graphs

In this paper a *graph* is an Eulerian 2-edge-colored undirected multi-graph with edges colored black or gray as in Fig. 1. A graph with equal multi-sets of black and gray edges is called *terminal*, and our goal is to transform a given graph into a terminal one using 2-breaks.

### **Definition 3**

(*2-break scenario*) A 2-break replaces two black edges $\{x_{1},x_{2}\}$ and $\{x_{3},x_{4}\}$ by either $\{x_{1},x_{3}\}$ and $\{x_{2},x_{4}\}$ or $\{x_{1},x_{4}\}$ and $\{x_{2},x_{3}\}$. A 2-break *scenario* of *length* *m* is a sequence of *m* 2-breaks transforming a graph into a terminal one.

### **Definition 4**

(*Eulerian graph and alternating cycle*) *G* is *Eulerian* if every vertex has equal black and gray degrees. A cycle is *alternating* if it is Eulerian. All use of the word *cycle* in this paper will be synonymous with alternating cycle.

Define a Maximum Alternating Edge-disjoint Cycle Decomposition (MAECD) of a graph *G* as a decomposition of *G* into a maximum number of edge-disjoint alternating cycles. Denote the size of a MAECD of *G* by *c*(*G*) and the number of its black edges by *e*(*G*). We make a distinction between simple cycles and circles (see Fig. 4 to see a simple cycle that is not a circle).

### **Definition 5**

(*simple cycle and circle*) A graph *G* is a *simple cycle* if the size of a MAECD, $c(G) = 1$. If in addition to that the black and gray degrees $deg^{b}(G,v)$ and $deg^{g}(G,v)$ are equal to 1 for every vertex *v*, then *G* is called a *circle*.

### Parsimonious 2-break scenarios

The problem of finding a minimum length (or *parsimonious*) 2-break scenario was treated in several unrelated settings using different terminology. Lemma 1 proven in “Proofs” section was treated in [20] where the authors also showed that finding a minimum length 2-break scenario is NP-hard due to the NP-hardness of finding a MAECD of a graph and provided a 7/4-approximation algorithm for finding this length. A variant of the problem for Eulerian digraphs where all the gray edges are loops was solved in [24].

#### **Lemma 1**

*(Bienstock and Günlük in* [20]) *The minimum length of a 2-break scenario on a graph* *G* *is* $d_{2b} (G) = e(G) - c(G)$.

Since finding a MAECD for a breakpoint graph is easy, Lemma 1 leads to a linear time algorithm for finding a parsimonious DCJ scenario [13]. The algorithm is based on Lemma 2 proven in “Proofs” section.

#### **Lemma 2**

*(Yancopoulos et al. in* [13]) *The minimum length of a DCJ scenario transforming genome* *A* *into* *B* *is equal to* $d_{2b} (G(A,B)) = e(G(A,B)) - c(G(A,B))$.

### Decomposition of a 2-break scenario

**Fig. 3:**
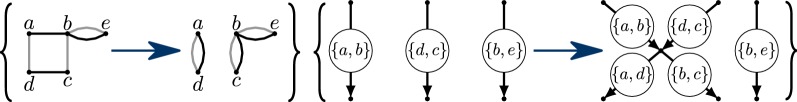
A 2-break $\{a,b\},\{d,c\}\rightarrow \{a,d\},\{b,c\}$ transforming a graph *G* into a terminal one is depicted on the left. A directed graph $\mathcal {D} (G,\rho )$ is obtained from $\mathcal {D} (G,\rho _{0})$ on the right for this scenario $\rho$ of length 1. The endpoints of the edges labeled $\{a,b\}$ and $\{d,c\}$ are merged and two new edges labeled $\{a,d\}$ and $\{b,c\}$ are introduced. $\mathcal {D} (G,\rho )$ has 2 connected components that correspond to the 2 simple cycles of *G*.

In this section we will show how a 2-break scenario $\rho$ of length *m* can be partitioned into subscenarios $\rho ^{1}, \ldots , \rho ^{k}$ and *G* can be decomposed into edge-disjoint Eulerian subgraphs $H^{1},\ldots , H^{k}$ where $\rho ^{i}$ is a scenario for $H^{i}$, and $k \ge e(G)-m$. We will use this decomposition in “[Sec Sec11]” section to show that $\varphi$-MCPS on a graph can be solved by solving $\varphi$-MCPS on its simple cycles. For a graph *G* and a 2-break scenario $\rho$ we define a directed 1-edge-colored edge-labeled graph $\mathcal {D} (G,\rho )$, akin to the *trajectory graph* introduced by Shao et al. [25]. Denote the sequence of the first *l* 2-breaks of $\rho$ by $\rho _{l}$ and the graph obtained from *G* after these 2-breaks by $G_{l}$. Define $\mathcal {D} (G,\rho _{0})$ in the following way: for each black edge *e* of *G* we have two new vertices connected by a directed edge labeled by *e* (see Fig. 3). For the *l*-th 2-break of $\rho$, $\{x_1, x_{2}\}, \{x_3, x_{4}\}\rightarrow \{x_1, x_{3}\}, \{x_2, x_{4}\}$, merge the endpoints of the edges labeled $\{x_1, x_{2}\}$ and $\{x_3, x_{4}\}$ in $\mathcal {D} (G,\rho _{l-1})$. Proceed by adding two new vertices to $\mathcal {D} (G,\rho _{l-1})$ and two edges labeled $\{x_1, x_{3}\}$ and $\{x_2, x_{4}\}$ from the merged vertex to the newly added ones to obtain $\mathcal {D} (G,\rho _{l})$. Continue until $\mathcal {D} (G,\rho _{m})$ is obtained, where *m* is the length of $\rho$, and denote it by $\mathcal {D} (G,\rho )$.

Shao et al. [25] characterize the connected components of a trajectory graph for a parsimonious scenario. Using similar techniques we prove the following lemma in “Proofs” section.

#### **Lemma 3**

*If* $\mathcal {D} (G,\rho )$ *has* *k* *connected components then* $\rho$ *can be partitioned into* *k* *subscenarios* $\rho ^{i}$ *and* *G* *can be partitioned into* *k* *edge-disjoint Eulerian subgraphs* $H^{i}$ *in such a way that* $\rho ^{i}$ *is a scenario for* $H^{i}$ *for every* $i\in \{1,\ldots ,k\}$. *If* $\rho$ *is parsimonious, then* $k=c(G)$ and $C(\rho ) = \{H^{1}, \ldots , H^{k}\}$ *is a* MAECD *of* *G*.

## Cost-constrained 2-breaks

In this section we outline our model for assigning costs to 2-breaks. We associate labels to both vertices and edges of a graph, and then describe a set $\mathcal {O}$ of *valid operations* of 2-breaks on labeled edges and edge-label changes. Our cost function is defined on $\mathcal {O}$. This model generalizes the labeled DCJ problems of [7, 11].

We will use letters *u*, *v*, *q*, *s* to denote vertices, letters *a*, *b*, *c*, *d* to denote vertex labels and *x*, *y*, *z*, *t* to denote edge labels. Given an alphabet of vertex labels $\Sigma _V$ and one of edge labels $\Sigma _E$, fix a subset $\mathcal {O}$ containing a set of tuples

- $\big((\{a,b\},x);(\{a,b\},y)\big)$ (called edge-label changes) and
- $\big ((\{a,b\},x),(\{c,d\},y);(\{a,c\},z),(\{b,d\},t)\big )$ (called 2-breaks on labels)

for $$a,b,c,d\in \Sigma _{V}$$ and $$x,y,z,t\in \Sigma _{E}$$.

Take a graph $G=(V,E)$, and its labeling $\lambda =(\lambda _{V},\lambda _{E})$ with $\lambda _V:V\rightarrow \Sigma _V$ and $\lambda _E: E\rightarrow \Sigma _E$. If $\mathcal {O}$ contains an edge-label change $\big ((\{a,b\},x);(\{a,b\},y)\big )$ and $(G,\lambda )$ contains an edge $\{u,v\}$ labeled *x* with vertices *u* and *v* labeled *a* and *b*, then the label of this edge can be changed into *y*. We call such a transformation of $(G,\lambda )$ an $\mathcal {O}$-change and denote it $(\{u,v\},x)\rightarrow (\{u,v\},y)$.

If $\mathcal {O}$ contains a 2-break on labels $\big ((\{a,b\},x),(\{c,d\},y);(\{a,c\},z),(\{b,d\},t)\big )$ and $(G,\lambda )$ contains two edges $\{u,v\}$ and $\{q,s\}$ labeled *x* and *y* respectively with vertices *u*, *v* and *q*, *s* labeled *a*, *b* and *c*, *d*, then a 2-break $\{u,v\},\{q,s\}\rightarrow \{u,q\},\{v,s\}$ can be performed on *G* with the labels of the new edges being *z* and *t*. We call such a transformation of $(G,\lambda )$ an $\mathcal {O}$-break and denote it $(\{u,v\},x),(\{q,s\},y)\rightarrow (\{u,q\},z),(\{v,t\},t)$.

An $\mathcal {O}$-*scenario* $\rho _{\mathcal {O}}$ for $(G,\lambda )$ is a sequence of $\mathcal {O}$-changes and $\mathcal {O}$-breaks transforming $(G,\lambda )$ into $(\overline{G},\overline{\lambda })$ such that $\overline{G}$ is terminal and its multi-sets of black and gray labeled edges are equal. The number of $\mathcal {O}$-breaks in $\rho _\mathcal {O}$ will be called the *2-break-length* of the scenario. If a $\rho _\mathcal {O}$ exists for $(G, \lambda )$, then $d_{\mathcal {O} b} (G,\lambda )$ denotes the minimum 2-break-length of an $\mathcal {O}$-scenario.

An $\mathcal {O}$-scenario does not necessarily exist for a given $(G,\lambda )$, however if it exists, then the inequality $d_{\mathcal {O} b} (G,\lambda )\ge d_{2b} (G)$ holds, where $d_{2b} (G)$ is the minimum length of a 2-break scenario on a graph *G*. In this paper we deal with the sets $\mathcal {O}$ that have the necessary operations to parsimoniously transform $(G,\lambda )$ into $(\overline{G},\overline{\lambda })$. We call these sets *p-sufficient*.

### **Definition 6**

*(p-sufficient* $\mathcal {O}$ *for* $(G,\lambda )$) A set $\mathcal {O}$ is *parsimonious-sufficient* or *p-sufficient* for $(G,\lambda )$ if we have $d_{\mathcal {O} b} (G,\lambda )=d_{2b} (G)$.

The cost function that we consider is $\varphi :\mathcal {O} \rightarrow \mathbb {R}_{+}$. The cost of an $\mathcal {O}$-scenario is the sum of the costs of its constituent operations. If $\mathcal {O}$ is p-sufficient for $(G,\lambda )$, then $MCPS _{\varphi }(G,\lambda )$ is the minimum cost of an $\mathcal {O}$-scenario of the 2-break-length equal to $d_{2b} (G)$, otherwise $MCPS _{\varphi }(G,\lambda )$ is $\infty$. We consider the following problem:

### Problem 1

($\varphi$-Minimum Cost Parsimonious Scenario or $\varphi$-MCPS)

$$\begin{aligned} \text {INPUT}{:}\;&\text { A graph }G,\text { and its labeling }\lambda .\\ \text {OUTPUT}{:}\;& MCPS _{\varphi }(G,\lambda ). \end{aligned}$$

### Examples of the cost-constrained DCJ problems in the literature

#### *Example 1*

(Minimum Local Parsimonious Scenario) In [11] we supposed the adjacencies of genome *A* to be partitioned into spatial regions represented by different colors. We then developed a polynomial time algorithm for finding a parsimonious DCJ scenario minimizing the number of rearrangements whose breakpoints appear in different regions. The problem as was stated in [11] differs slightly from $\varphi$-MCPS, since in that study we do not have colors for the adjacencies of genome *B*. We can bridge this gap as follows.

Edge labels $\Sigma _{E}=\Sigma _c\cup \{\tau \}$ are the colors representing the different spatial regions of a genome plus an additional terminal label $\tau$. There is a single vertex label $\Sigma _V=\{a\}$. $\mathcal {O}$ contains 2-breaks on labels $\big ((\{a,a\},x),(\{a,a\},y);(\{a,a\},x),(\{a,a\},y)\big )$ for $x,y\in \Sigma _c$, and edge-label changes $\big ((\{a,a\},x);(\{a,a\},\tau )\big )$ for $x\in \Sigma _c$. The cost $\varphi _{c}$ of a 2-break on labels in $\mathcal {O}$ is 0 if the 2 labels being replaced are equal and 1 otherwise. The cost of a edge-label change is 0.

In [11] we presented an $O(n^4)$ time algorithm solving $\varphi _{c}$-MCPS for a labeled breakpoint graph with the gray edges labeled by $\tau$. In [12] we demonstrated that finding a minimum cost $\mathcal {O}$ scenario for such a breakpoint graph, when the parsimonious criteria is disregarded, is NP-hard. We proposed an algorithm that is exponential in the number of colors but not in the number of genes.

In “[Sec Sec15]” section we use the same $\mathcal {O}$, fix a symmetric function $\Phi :\Sigma ^2\rightarrow \mathbb {R_{+}}$, and define $\varphi _{f}\big ((\{a,a\},x),(\{a,a\},y);(\{a,a\},x),(\{a,a\},y)\big )=\Phi (x,y)$. This drastically enhances the model introduced in [11] as now rearrangements whose breakpoints appear in the same region can have non-zero costs. In “[Sec Sec13]” section we provide an $O(n^5)$ time algorithm solving the generalized problem of $\varphi _{f}$-MCPS for a labeled breakpoint graph.

#### *Example 2*

(DCJ weighted by Hi-C) In [10] we weighted each DCJ by the value taken directly from the Hi-C contact map. In this model every intergenic region of genome *A* gets assigned an interval corresponding to its genomic coordinates on a chromosome. The *weight* of a DCJ acting on two intergenic regions is then equal to the average Hi-C value for their corresponding intervals. In [10] we presented an algorithm greedily maximizing the weight of a parsimonious scenario and found that the obtained weight is significantly higher than the weight of a random parsimonious scenario.

Edge labels are the genomic intervals corresponding to the intergenic regions of a genome *A* plus an additional terminal label. There is a single vertex label $\Sigma _V=\{a\}$. $\mathcal {O}$ stays as in Example 1. $\Phi _{HiC}(x,y)$ on two genomic intervals is their average Hi-C value. The problem that maximizes Hi-C values can be easily transformed into a minimization problem by setting the cost of a 2-break on labels $\big ((\{a,a\},x),(\{a,a\},y);(\{a,a\},x),(\{a,a\},y)\big )$ to $\Phi _{\max }-\Phi _{HiC}(x,y)$, where $\Phi _{\max }$ is the maximum $\Phi _{HiC}(x,y)$ over all *x*, *y*.

In [10] the optimality of the proposed greedy algorithm was not discussed, but our work presented in “[Sec Sec15]” section of this paper provides us with a polynomial time algorithm for solving this problem exactly.

#### *Example 3*

(Sorting by wDCJs and indels in intergenes) Bulteau et al.  [7] introduced a problem where adjacencies of genomes are labeled with their genetic length (number of nucleotides). A *wDCJ* is a DCJ that preserves the sum of the genetic lengths of the adjacencies and an *indel* $\delta$ increases or decreases the genetic length of an adjacency by $\delta$. The cost of a wDCJ is 0 and the cost of an indel $\delta$ is $|\delta |$. A scenario of wDCJs and indels for $(G,\lambda )$ is said to be *valid* if its wDCJ-length is $d_{2b} (G)$. The paper presents an $O(n\log n)$ algorithm for finding a minimum cost scenario among the *valid* ones, for the genomes with circular chromosomes and *n* genes.

Translating this into our formalism yields the following $\varphi$-MCPS problem. Edge labels are the natural numbers, there is a single vertex label, and $\mathcal {O}$ contains 2-breaks on labels $((\{a,a\},w_{1}),(\{a,a\},w_{2});(\{a,a\},w_{3}),(\{a,a\},w_{4})\big )$ for $w_{i}\in \Sigma _{E}$ satisfying $w_{1}+w_{2}=w_{3}+w_{4}$. $\mathcal {O}$ also contains edge-label changes $\big ((\{a,a\},w_1);(\{a,a\},w_2)\big )$ for $w_{i}\in \Sigma$. $\mathcal {O}$ is p-sufficient for any $(G,\lambda )$ since *G* can be first transformed into a terminal graph using any parsimonious 2-break scenario and then its labels can be adjusted. The cost $\varphi _{l}$ of a 2-break on labels is 0 and the cost $\varphi _{l}$ of a edge-label change $\big ((\{a,a\},w_1);(\{a,a\},w_2)\big )$ is $|w_{1}-w_{2}|$.

In [7] the authors presented an $O(r\log r)$ time algorithm for solving $\varphi _{l}$-MCPS on a circle with *r* vertices. Combining this algorithm with our results from “[Sec Sec13]” section gives an algorithm solving $\varphi _{l}$-MCPS in $O(n^3)$ time for a labeled breakpoint graph. The ILP defined in “[Sec Sec11]” section solves $\varphi _{l}$-MCPS for any labeled graph.

#### Example 4

(wDCJ-dist) Fertin et al. [6] treated a problem wDCJ-dist where wDCJs without indels are allowed, and the sums of the genetic lengths of the adjacencies of two genomes are equal.

In this case we keep the same $\Sigma _{E}, \Sigma _{V}$ and $\mathcal {O}$ as in Example 3 except that the edge-label changes are excluded from $\mathcal {O}$. A labeled graph is said to be *balanced* if the sums of the labels of black and gray edges are equal. wDCJ-dist is the problem of finding $d_{\mathcal {O} b}$ for a balanced graph whose connected components are circles. The authors show that wDCJ-dist is strongly NP-complete. However they also prove that $d_{\mathcal {O} b} (O,\lambda )=d_{2b} (O)$ for a balanced circle *O* and that $\mathcal {O}$ is p-sufficient for a graph whose connected components are balanced circles.

#### Example 5

Although ignored in the previous examples, the weighting of operations based on only the vertices is also possible under our framework. For example, take $\Sigma _{E}=\{\tau \}$, $\Sigma _{V}=\mathbb {N}$, $\mathcal {O}$ containing 2-breaks on labels $((\{a,b\},\tau ),(\{c,d\},\tau );(\{a,c\},\tau ),(\{b,d\},\tau )\big )$ and any cost function $\varphi _{v}:\mathcal {O} \rightarrow \mathbb {R}_{+}$. The costs of the 2-breaks on labels in $\mathcal {O}$ could be a function of the genomic coordinates of the participating gene extremities.

Note that the set $\mathcal {O}$ is implicit, rather than explicit. In Example 3, $\mathcal {O}$ would be too large to represent explicitly since every pair of genetic lengths for every pair of edges would exist. For all practical uses that we know of to date, membership in $\mathcal {O}$ can be computed in constant time.

## $\varphi$-MCPS for a graph

### **Theorem 1**

*Denote the* $\varphi$-*cost* *of a* MAECD *as the sum of the* $MCPS_{\varphi }$ *on its cycles*. $MCPS _{\varphi }$ *for a graph is equal to the minimum* $\varphi$-*cost of its* MAECD.

### *Proof*

For a cycle *S* of a labeled graph $(G,\lambda )$, $\lambda ^{S}$ denotes the labeling of *S* according to $\lambda$. We suppose that $min(\emptyset )=\infty$ and prove the following:

$$\ MCPS_{\varphi } (G,\lambda ) = min\left\{ {\sum\limits_{{S \in C}} {MCPS_{\varphi } } (S,\lambda ^{S} )~|~C~{\text{is a}}~MAECD~{\text{of}}~G} \right\}.{\text{ }}$$

Suppose that there exists a MAECD *C* of *G* consisting of the simple cycles for which $\mathcal {O}$ is p-sufficient. For every $S\in C$ take an $\mathcal {O}$-scenario $\rho _{\mathcal {O}}^{S}$ of cost $MCPS _{\varphi }(S,\lambda ^S)$ and 2-break-length $d_{2b} (S)$. By performing these scenarios one after another we obtain an $\mathcal {O}$-scenario $\rho _{\mathcal {O}}$ for $(G,\lambda )$ of 2-break-length $\sum _{S\in C}d_{2b} (S)=d_{2b} (G)$ and of cost $\sum _{S\in C}MCPS _\varphi (S,\lambda ^{S})$. This means that $MCPS _{\varphi }(G,\lambda )\le \sum _{S\in C}MCPS _\varphi (S,\lambda ^{S})$.

On the other hand, suppose that $\mathcal {O}$ is p-sufficient for $(G,\lambda )$ and take an $\mathcal {O}$-scenario $\rho _{\mathcal {O}}$ for $(G,\lambda )$ of length $d_{2b} (G)$. For $\rho$, a 2-break scenario obtained from $\rho _{\mathcal {O}}$ when the labels are neglected, a decomposition $C(\rho )$ corresponding to $\rho$ is a MAECD of *G* due to Lemma 3. A subsequence $\rho ^{S}_{\mathcal {O}}$ of $\rho _{\mathcal {O}}$, consisting of the operations acting on the edges of a cycle $S\in C(\rho )$, is an $\mathcal {O}$-scenario for $(S,\lambda ^{S})$ of 2-break-length $d_{2b} (S)$. A sequence of operations $\hat{\rho }_{\mathcal {O}}$ obtained by performing the subsequences $\rho ^{S}_{\mathcal {O}}$ one after another for each $S\in C(\rho )$ is an $\mathcal {O}$-scenario for $(G,\lambda )$. By construction the 2-break-length of $\hat{\rho }_{\mathcal {O}}$ is equal to the 2-break-length of $\rho _{\mathcal {O}}$. The costs of $\rho _{\mathcal {O}}$ and $\hat{\rho }_{\mathcal {O}}$ are also equal, as they consist of exactly the same operations that are performed in different orders, thus the cost of $\rho _{\mathcal {O}}$ is greater or equal to $\sum _{S\in C(\rho )}MCPS _{\varphi }(S,\lambda ^{S})\ge min\big \{\sum _{S \in C}MCPS _\varphi (S,\lambda ^{S})~\big |~C~\text {is a}~MAECD~\text {of}~G\big \}$. $\square$

Take the set $\mathcal {S}$ of simple labeled cycles of $(G,\lambda )$. If one can solve $\varphi$-MCPS for every $S\in \mathcal {S}$, then Theorem 1 provides a straightforward way to solve $\varphi$-MCPS for $(G,\lambda )$ as a set packing problem. First compute *c*(*G*) by solving the ILP in the left column. Then proceed by solving the other ILP to compute $MCPS_{\varphi }(G,\lambda).$

$$\begin{array}{ll} {\begin{array}{ll}{\text{Maximize }}&{\sum\nolimits_{S\in {\mathcal {S}}}} \, x_{S}\\ {\text{Subject to}}&{\sum\nolimits_{S:e\in S}} x_{S}\leq 1\ {\text{for each edge }} e {\text{ of }} G\\ &{\text{and}} \ x_{S}\in\{0,1\} \text{ for simple cycle } S\in {\mathcal {S}}.\end{array} }&\quad{\begin{array}{ll} {\text{Minimize }}&{\sum\nolimits_{S\in {\mathcal {S}}}}\, x_{S}MCPS_{\varphi}(S,\lambda^{S})\\ \text{Subject to }&{\sum\nolimits_{S:e\in S}} \, x_{S}\leq 1{\text{ for each edge }} e {\text{ of }} G,\\ &{\sum\nolimits_{S\in {\mathcal {S}}}}\, x_{S} = c(G)\\ &{\text{and }} \ x_{S}\in\{0,1\} \text{ for simple cycle } S\in {\mathcal {S}}.\end{array}}\end{array}$$

There exists an algorithm efficiently listing all the simple cycles of an undirected 1-edge-colored graph [26], however we are unaware of a similar result for the 2-edge-colored graphs. Computing *c*(*G*) is an APX-hard problem [27] and the size of $\mathcal {S}$ may be exponential in the size of *G*, which might make these ILPs intractable in general. For graphs representing genomes with duplicate genes, the number of simple cycles can grow exponentially as a function of the number of duplicate genes. For breakpoint graphs, however, the number grows quadratically and *c*(*G*) can be found in linear time.

## $\varphi$-MCPS for a simple cycle

The decomposition theorem of “[Sec Sec11]” section reduces the computation of $\varphi$-MCPS on a graph to the computation of $\varphi$-MCPS on a simple alternating cycle. In this section we further decompose the problem into simpler versions of cycles, called circles, which are alternating cycles that contain a vertex only once.

**Fig. 4:**
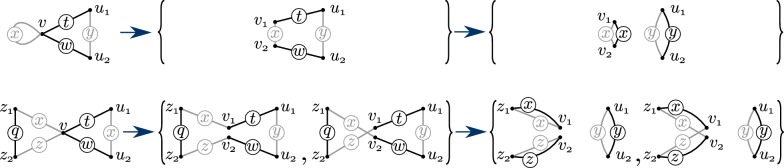
Two simple cycles having a vertex *v* of degree two are depicted in the first column. Their sets of corresponding circles obtained by splitting *v* into $v_{1}$ and $v_{2}$ are depicted in the second column. This set is of size 1 for the upper simple cycle containing the gray loop $\{v,v\}$, and of size 2 for the lower simple cycle. An $\mathcal {O}$-scenario for a simple cycle yields a scenario of the same cost and length transforming the graphs in the second column to those that become terminal once $v_1$ and $v_2$ are merged. One possible outcome of such a scenario is presented in the third column

Denote $deg_{2} (G)$ for a graph *G* as the number of vertices with black and gray degree equal to two. It is easy to check that $deg^{b}(S,v) = deg^{g}(S,v) \le 2$ for any vertex *v* of a simple cycle *S*. If $deg_{2} (S)=0$, then *S* is a circle. See the first column of Fig. 4 for examples of simple cycles that are not circles.

Take a simple labeled cycle $(S,\lambda )$ and denote $S_{0}$ as $\{(S,\lambda )\}$. Choose a vertex *v* of degree two in *S* and replace it by two vertices $v_{1},v_{2}\notin V$ labeled by the same label as *v*. If *v* is incident to a gray loop, then split it into two vertices $v_{1}$ and $v_{2}$, as depicted on the top row of Fig. 4, to obtain a set $S_{1}$ consisting of a single simple cycle. Otherwise split it into two vertices, as depicted on the bottom row of Fig. 4, to obtain a set $S_{1}$ consisting of two simple cycles.

Simple labeled cycles in $S_{1}$ share the same set of vertices of degree two. Choose such a vertex and split it simultaneously in all the cycles in $S_{1}$ as previously to obtain a set $S_{2}$ of at most 4 simple labeled cycles sharing the same set of vertices and the same multi-set of labeled black edges. Continue this procedure until the set $circ(S,\lambda )=S_{deg_{2} (S)}$ of the labeled circles is obtained.

### **Theorem 2**

$MCPS _\varphi$ *for a simple cycle* $(S,\lambda )$ *is equal to the minimum of the* $MCPS _\varphi$ *among the circles in* $circ(S,\lambda )$.

### *Proof*

First we prove that $MCPS _\varphi (S,\lambda )=min\{MCPS _\varphi(H,\mu )|~(H,\mu )\in ~S_{1}\}$. Labeled graphs in $S_{1}$ are obtained by splitting a vertex *v* of degree 2 into vertices $v_{1}$ and $v_{2}$. For a labeled graph $(H,\mu )$ on vertices $V\cup \{v_{1},v_{2}\}\setminus \{v\}$ denote $r_{g}(H,\mu )$ as the labeled graph obtained from $(H,\mu )$ by reversing the split, that is, by merging the vertices $v_{1}$ and $v_{2}$ into *v*.

Choose $(\hat{S},\hat{\lambda })\in ~S_{1}$. By construction $r_{g}(\hat{S},\hat{\lambda })=(S,\lambda )$. Denote $r_{v}(v_{1})=r_{v}(v_{2})=v$, and $r_{v}(u)=u$ for $u\in V$. For an edge *f* of $(\hat{S},\hat{\lambda })$ joining vertices *q* and *s*, the edge $r_{e}(f)=\{r_{v}(q),r_{v}(s)\}$ is present in $(S,\lambda )$ and has the same label as *f*. $r_{e}$ defines a bijection between the labeled edges of $(S,\lambda )$ and $(\hat{S},\hat{\lambda })$ and thus between $\mathcal {O}$ operations on these graphs. This means that an operation in $\mathcal {O}$ transforming $(\hat{S},\hat{\lambda })$ into some $(\hat{S}',\hat{\lambda }')$ transforms $(S,\lambda )$ into $r_{g}(\hat{S}',\hat{\lambda }')$, and an operation in $\mathcal {O}$ transforming $(S,\lambda )$ into some $(S',\lambda ')$ transforms $(\hat{S},\hat{\lambda })$ into $(\hat{S}',\hat{\lambda }')$ such that $r_{g}(\hat{S}',\hat{\lambda }')=(S',\lambda ')$.

Thus for an $\mathcal {O}$-scenario of $(\hat{S},\hat{\lambda })$ there exists an $\mathcal {O}$-scenario of the same $\varphi$ cost and the same 2-break-length for $(S,\lambda )$. On the other hand, an $\mathcal {O}$-scenario for $(S,\lambda )$ provides us with a sequence $\rho$ of $\mathcal {O}$ operations of the same $\varphi$ cost and the same 2-break-length transforming $(\hat{S},\hat{\lambda })$ into $(\overline{S},\overline{\lambda })$ for which $r_{g}(\overline{S},\overline{\lambda })$ is a terminal graph.

If $S_{1}$ is of size 1, then there is a single choice for $(\overline{S},\overline{\lambda })$ (see the right upper corner of Fig. 4) and it is itself terminal. If $S_{1}$ is of size 2, then there are two options for $(\overline{S},\overline{\lambda })$ (see the right bottom corner of Fig. 4). Either $(\overline{S},\overline{\lambda })$ is already terminal, or the sequence $\rho$ of $\mathcal {O}$ operations transforming $(\hat{S},\hat{\lambda })$ into $(\overline{S},\overline{\lambda })$ transforms the second graph in $S_{1}$ into a terminal one.

Now we prove that $MCPS _\varphi(S,\lambda ) = min\{MCPS _\varphi(O,\lambda )|~(O,\lambda )\in ~circ(S,\lambda )\}$, which is clearly true for $deg_{2} (S)=0$. Suppose this to be true for $deg_{2} (S)<t$. We prove it for $deg_{2} (S)=t$ by induction. For $(\hat{S},\hat{\lambda })\in S_{1}$ one has $deg_{2} (\hat{S}) = t-1$, so using the inductive hypothesis we have that $MCPS _\varphi(\hat{S},\hat{\lambda })$ is equal to $min\{MCPS _\varphi(O,\lambda )|~(O,\lambda )\in ~circ(\hat{S},\hat{\lambda })\}$. We have already proven that $MCPS _\varphi(S,\lambda ) = min\{MCPS _\varphi(H,\mu )|~(H,\mu )\in ~S_{1}\}$, and by construction we know that $circ(S,\lambda ) = \cup _{(H,\mu )\in S_{1}} circ(H,\mu )$. These combine to imply that the theorem is true for $deg_{2} (S)=t$. $\square$

## $\varphi$-MCPS for a breakpoint graph

In this section we suppose that there exists an algorithm for computing $MCPS _\varphi$ on a labeled circle (e.g. the algorithm of “[Sec Sec15]” section). Using this algorithm as a subroutine we will construct an algorithm for finding $MCPS _\varphi$ for a labeled breakpoint graph. This is a generalization of the work first presented in [11].

Take genomes *A* and *B* partitioned into *n* genes where each gene occurs exactly once in each genome, and a labeling $\lambda$ of a breakpoint graph *G*(*A*, *B*). For all the vertices $v\ne \circ$ we have $deg^{g}(G(A,B),v)=deg^{b}(G(A,B),v)=1$. Thus, if there is a circle in *G*(*A*, *B*) containing an edge then this circle is the only simple cycle containing this edge. This means that every MAECD of *G*(*A*, *B*) includes all of its circles. These set aside we are left with $G(A,B)'$, which is a union of alternating paths starting and ending at $\circ$ with end edges of the same color. If this color is black we call the path *AA*, and *BB* otherwise.

We proceed by constructing a complete weighted bipartite graph *H* having the *AA* and *BB* paths of $G(A,B)'$ as vertices. Every simple cycle of $G(A,B)'$ is a union of an *AA* path and a *BB* path. To each edge joining these paths in *H* we assign weight equal to $MCPS _\varphi$ for a union of these paths. A MAECD of $G(A,B)'$ corresponds to a maximum matching of *H* and every such matching corresponds to a MAECD of $G(A,B)'$. Denote $\lambda '$ as the labeling of $G(A,B)'$ according to $\lambda$. Using Theorem 1 we obtain that $MCPS _\varphi(G(A,B)',\lambda ')$ is equal to the minimum weight of a maximum matching of *H*. There is an equal number *p* of *AA* and *BB* paths. Let *P* denote the total number of edges in $G(A,B)'$. Using this notation we obtain the following lemma proven in “Proofs” section.

### **Lemma 4**

*For a function* *f* *and an* *O*(*f*(*r*)) *time algorithm for* $\varphi$-MCPS *on a labeled circle on* *r* *vertices, there exists an* ${O(p^2f(P)+p^3+f(n))}$ *time algorithm for* $\varphi$-MCPS *on a labeled breakpoint graph. If* $f(r)=O(r^t)$ *for some constant* $t\ge 1$, *then* $\varphi$-MCPS *on a labeled breakpoint graph can be solved in* $O(pP^t+p^3+n^t)$ *time*.

Both *p* and *P* are *O*(*n*), thus Lemma 4 leads to the following theorem.

### **Theorem 3**

*Given a constant* $t\ge 2$ *and an* $O(r^t)$ *time algorithm for* $\varphi$-MCPS *on a labeled circle on* *r* *vertices*, $\varphi$-MCPS *on a labeled breakpoint graph can be solved in* $O(n^{t+1})$ *time*.

### **Corollary 1**

*Using the* $O(r^4)$ *algorithm from* “[Sec Sec15]” section *we obtain an* $O(n^{5})$ *algorithm for solving* $\varphi _{f}$-MCPS *on a labeled breakpoint graph with fixed labels.*

### **Corollary 2**

*Using the* $O(r\log r)$ *algorithm from* [7] *for the* Sorting by wDCJs and indels in intergenes *problem on a circle (see Example* 3*), we obtain an* $O(n^3)$ *algorithm for solving the problem on a breakpoint graph*.

## $\alpha$-approximation for $\varphi$-MCPS

Theorems 1 and 2 demonstrate how $\varphi$-MCPS for any labeled graph can be solved if one is able to solve $\varphi$-MCPS for a labeled circle. This is exploited in Theorem 3 to solve $\varphi$-MCPS for a breakpoint graph. Analogous results proven in “Proofs” section hold if instead of an exact algorithm one has an $\alpha$-approximation for $\varphi$-MCPS for a labeled circle.

### **Lemma 5**

*For a constant* $t\ge 2$ *and an* $O(r^t)$ *time* $\alpha$*-approximation algorithm for* $\varphi$-MCPS *on a labeled circle on* *r* *vertices, there exists an* $O(n^{t+1})$ *time* $\alpha$-*approximation algorithm for* $\varphi$-MCPS *on a labeled breakpoint graph.*

## $\varphi _{f}$-MCPS for a circle with fixed labels

Here we define $\varphi _{f}$-MCPS, a particular instance of a $\varphi$-MCPS problem, and solve it for a circle. $\varphi _{f}$-MCPS generalizes our previous work presented in Examples 1 and 2. For a set $\Sigma _{V}=\{a\}$ of vertex labels and a set $\Sigma _{E}=\Sigma \cup \{\tau \}$ of edge labels, define a set $\mathcal {O}$ consisting of 2-breaks on labels $\big ((\{a,a\},x),(\{a,a\},y);(\{a,a\},x),(\{a,a\},y)\big )$ for $x,y\in \Sigma$, and edge-label changes $\big ((\{a,a\},x);(\{a,a\},\tau )\big )$ for $x\in \Sigma$. Fix a symmetric function $\Phi :\Sigma ^{2}\rightarrow \mathbb {R}_{+}$ and define a $\varphi _{f}$ cost of a 2-break on labels $\big ((\{a,a\},x),(\{a,a\},y);(\{a,a\},x),(\{a,a\},y)\big )$ to be $\Phi (x,y)$ and a $\varphi _{f}$ cost of an edge-label change $\big ((\{a,a\},x);(\{a,a\},\tau )\big )$ to be 0. We will provide a polynomial time algorithm for $\varphi _{f}$-MCPS on a labeled circle with the gray edges labeled by a terminal label $\tau$.

**Fig. 5:**
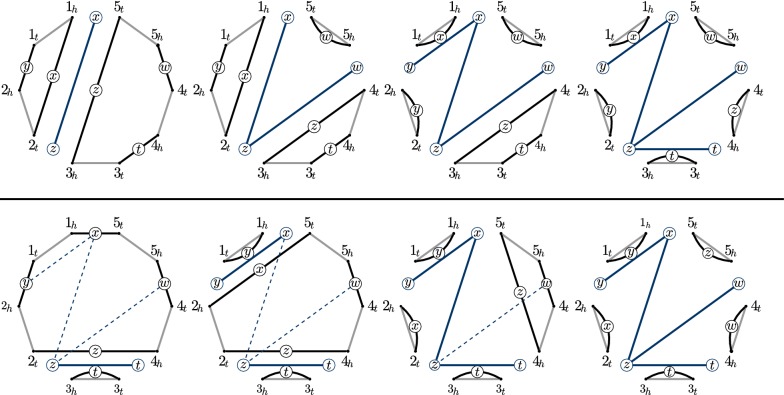
On the top: 4 steps of a parsimonious $\mathcal {O}$-scenario for a circle are depicted together with each $\mathcal {T}$ corresponding to the scenario at that point colored in blue. Vertices of $\mathcal {T}$ are superimposed on the corresponding edges of a circle providing their circular embedding. All of the $\mathcal {T}$ are planar trees. On the bottom: for a given planar tree $\mathcal {T}$ (dashed blue) we provide a scenario $\rho _{\mathcal {O}}$ such that $\mathcal {T} (\rho _{\mathcal {O}})=\mathcal {T}$

Without loss of generality we can suppose that all of the black edges of a circle have different labels; if two edges are labeled with the same label *x*, then we simply replace one of these labels with a new label $\hat{x}$ and set $\hat{\Phi }(\hat{x},y)=\Phi (x,y)$ and $\hat{\Phi }(y,z)=\Phi (y,z)$ for $y,z\in \Sigma$.

For a labeled circle having *r* black edges, define a set $V_{\Sigma }$ of *r* vertices corresponding to their labels. For an $\mathcal {O}$-scenario $\rho _{\mathcal {O}}$ we define a 1-edge-colored undirected graph $\mathcal {T} (\rho _{\mathcal {O}})$ with vertices $V_{\Sigma }$ and an edge $\{x,y\}$ for every $\mathcal {O}$-break in $\rho _{\mathcal {O}}$ replacing the black edges labeled with *x* and *y* (see Fig. 5). The *cost* of an edge $\{x,y\}$ is defined to be $\Phi (x,y)$ and the cost of a graph $\mathcal {T} (\rho _{\mathcal {O}})$ is the sum of the costs of its edges. The costs of $\rho _{\mathcal {O}}$ and $\mathcal {T} (\rho _{\mathcal {O}})$ are equal by construction.

Fix a circular embedding of $V_{\Sigma }$ respecting the order of the black edges on the labeled circle (see Fig. 5). A graph with vertices $V_{\Sigma }$ is said to be *planar on the circle* if none of its edges cross in this embedding. We prove Lemma 6 in “Proofs” section linking planar trees and parsimonious scenarios.

### **Lemma 6**

*If* $\rho _{\mathcal {O}}$ *is a minimum 2-break-length* $\mathcal {O}$-*scenario for a labeled circle* $(O,\lambda )$, *then* $\mathcal {T} (\rho _{\mathcal {O}})$ *is a planar tree on* $(O,\lambda )$. *In addition to that, for a planar tree* $\mathcal {T}$ *on* $(O,\lambda )$ *there exists an* $\mathcal {O}$-*scenario* $\rho _{\mathcal {O}}$ *such that* $\mathcal {T} (\rho _{\mathcal {O}})=\mathcal {T}$.

Farnoud and Milenkovic in [19] provide a dynamic programming algorithm for finding a minimum cost planar tree on a circle. In “Proofs” section their proof for a following lemma is given which, together with Lemma 6, leads to Theorem 4.

### **Lemma 7**

(Farnoud and Milenkovic in [19]) *A minimum cost planar tree on a circle can be found in* $O(r^4)$ *time, where* *r* *is the number of vertices of a tree*.

### **Theorem 4**

$\varphi _{f}$-MCPS *for a labeled circle on* *r* *vertices can be solved in* $O(r^4)$ *time*.

## Conclusions and future directions

### Practical matters

Our algorithm for $\varphi _{f}$-MCPS on a breakpoint graph with fixed labels has a running time of $O(n^5)$ in the worst case. Note that the running time is dominated, however, by the maximum bipartite matching step in “[Sec Sec13]” section. The size of the bipartite graph is determined by the number of *AA* and *BB* paths which is bounded by the maximum number of chromosomes *m* for the two species. Thus using Lemma 4 we know that the algorithm scales like $O(mn^4)$ on biological data. For the same reason our algorithm for Sorting by wDCJs and indels in intergenes  [7] on a breakpoint graph scales like $O(m^{2}n\log {n}+m^3)$ instead of $O(n^3)$ on biological data. Further, *n* is the number of syntenic blocks—and not literally the genes as we call them. Our analyses of *Drosophila* genomes yield no *AA* and *BB* paths, and less than 100 blocks [10]. Our analysis of Human and Mouse genomes yields between 250 and 800 syntenic blocks, depending on the parameters given to OrthoCluster [28].

For graphs with higher degree nodes, like those graphs that represent genomes with duplicated genes, the number of simple cycles can grow rapidly. Although this relationship is beyond the scope of this work, we expect that fixed parameter algorithms could be developed to handle biological data in the future.

### Future direction

Our cost framework is liberal, and in our examples we have explored only a small portion of its capacities. Edges can be labeled by more complex objects such as vectors or trees. The cost can be a function of a combination of the edge and vertex labels. We hope that a closer study of the graph $\mathcal {D} (G,\rho )$ from “Decomposition of a 2-break scenario” section will lead to polynomial time algorithms for $\varphi$-MCPS on circles for a large family of cost functions. Once the set of scenarios for a circle is better understood, one could address the problems of counting and sampling the $\varphi$-MCPS scenarios.

While all of our results apply to genomes with insertions or deletions of single genes, further study is required in order to increase efficiency on genomes with duplicate genes.

Our assumption of “minimum evolution” may not always be true as an actual evolutionary scenario might be non-parsimonious [29]. The Minimum Cost Scenario (MCS) problem of finding a minimum cost scenario among all the possible scenarios has already been studied for a couple of fairly simple cost functions [6, 12] and proven to be NP-hard in both of these cases. However, as we have shown in [12], computationally tractable algorithms can still be implemented for certain NP-hard MCS problems. An intermediate problem between MCPS and MCS could be the one of finding a minimum cost scenario among the scenarios of a prescribed length.

## Proofs

### Lemma 1

#### **Lemma**

(Bienstock and Günlük in [20]) *The minimum length of a 2-break scenario on a graph* *G* *is* $d_{2b} (G) = e(G) - c(G)$.

#### *Proof*

A 2-break can increase the size of a MAECD by at most 1 and the size of a MAECD of a terminal graph is *e*(*G*). This leads to an inequality $d_{2b} (G)\ge e(G)-c(G)$.

In this paragraph the *length* of a cycle will be its number of black edges. For any cycle *c* of length $l > 1$ there is a 2-break transforming *c* into a union of length 1 and length $l-1$ cycles. This way we obtain a scenario of length $l-1$ for *c*, and can transform every cycle of a MAECD of *G* independently, obtaining a 2-break scenario of length $e(G)-c(G)$. Thus, $d_{2b} (G)\le e(G)-c(G)$. $\square$

### Lemma 2

#### **Lemma**

(Yancopoulos et al. in [13]) *The minimum length of a DCJ scenario transforming genome* *A* *into* *B* *is equal to* $d_{2b} (G(A,B)) = e(G(A,B)) - c(G(A,B))$.

#### *Proof*

*G*(*A*, *B*) is constructed in such a way that for every DCJ $A\rightarrow A'$ the transformation $G(A,B)\rightarrow G(A',B)$ is a 2-break. Notably, a DCJ $\{a,b\}\rightarrow \{a\},\{b\}$ results in a transformation $\{a,b\},\{\circ ,\circ \}\rightarrow \{a,\circ \},\{b,\circ \}$, as the construction of a breakpoint graph guarantees that there are enough black loops $\{\circ ,\circ \}$ to realize such a 2-break. For any 2-break $G(A,B)\rightarrow G'$ with $G'\ne G(A,B)$ there exists a DCJ $A\rightarrow A'$ such that $G(A',B)=G'$. Since *G*(*B*, *B*) is terminal, it follows that the minimum length of a scenario transforming *A* into *B* is $d_{2b} (G(A,B))$ and we conclude using Lemma 1. $\square$

### Lemma 3

#### **Lemma**

*If* $\mathcal {D} (G,\rho )$ *has* *k* *connected components then* $\rho$ *can be partitioned into* *k* *subscenarios* $\rho ^{i}$ *and* *G* *can be partitioned into* *k* *edge-disjoint Eulerian subgraphs* $H^{i}$ *in such a way that* $\rho ^{i}$ *is a scenario for* $H^{i}$ *for every* $i\in \{1,\ldots ,k\}$. *If* $\rho$ *is parsimonious, then* $k=c(G)$ *and* $C(\rho ) = \{H^{1}, \ldots , H^{k}\}$ *is a* MAECD *of* *G*.

#### *Proof*

Take a connected component *C* of $\mathcal {D} (G,\rho )$. It has an equal number of vertices of indegree 0 and vertices of outdegree 0. Its edges incident to the vertices of indegree 0 are labeled with the black edges of *G* and its edges incident to the vertices of outdegree 0 are labeled with the gray edges of *G*. Together these labels define a subgraph *H* of *G* that we will prove to be Eulerian.

Define $C_{l}$ to be a subgraph of $\mathcal {D} (G,\rho _{l})$ consisting of its connected components containing the vertices of indegree 0 of *C*. This way $C_{m}=C$. Define $H_{l}$ to be a subgraph of $G_{l}$ containing the gray edges of *H* and the black edges of $G_{l}$ labeling the edges of $C_{l}$ incident to the vertices of outdegree 0. This way $H_{0}=H$ and $H_{m}$ is a terminal graph.

We prove that *H* is Eulerian by induction. $H_{m}$ is Eulerian as it is terminal. Suppose that $H_{l}$ is Eulerian. By construction the two edges of $G_{l}$ replaced by the *l*-th 2-break of $\rho$ either both belong to $H_{l-1}$ or both are outside of $H_{l-1}$. In the first case, $H_{l}$ is obtained from $H_{l-1}$ via a 2-break and as $H_{l}$ is Eulerian this means that $H_{l-1}$ is also Eulerian. In the second case, $H_{l}=H_{l-1}$, thus the latter stays Eulerian. Thus $H=H_{0}$ is Eulerian and we obtain a subsequence of $\rho$ that is a scenario for *H*.

$\mathcal {D} (G,\rho _0)$ has *e*(*G*) connected components. The *l*-th 2-break of $\rho$ merges two vertices of $\mathcal {D} (G,\rho _{l-1})$, thus reduces the number of the connected components by at most 1. This means that the number *k* of the connected components of $\mathcal {D} (G,\rho )$ is greater or equal to $e(G)-m$.

If $\rho$ is parsimonious, then its length *m* is $e(G) - c(G)$ using Lemma 1. This means that $k\ge c(G)$ and *G* can be partitioned into *k* edge-disjoint Eulerian subgraphs. Due to the maximality of *c*(*G*), we have that $k=c(G)$ and all of the obtained edge-disjoint Eulerian subgraphs of *G* are simple cycles. $\square$

### Lemma 4

#### **Lemma**

*For a function* *f* *and an* *O*(*f*(*r*)) *time algorithm for* $\varphi$-MCPS *on a labeled circle on* *r* *vertices, there exists an* ${O(p^2f(P)+p^3+f(n))}$ *time algorithm for* $\varphi$-MCPS *on a labeled breakpoint graph. If* $f(r)=O(r^t)$ *for some constant* $t\ge 1$, *then* $\varphi$-MCPS *on a labeled breakpoint graph can be solved in* $O(pP^t+p^3+n^t)$ *time*.

#### *Proof*

The $p^2$ edges of a bipartite graph *H* can be weighted in $O(p^2f(P))$ time due to Theorem 2 and the fact that the simple cycles of *G*(*A*, *B*) have at most 1 vertex of degree 2. A minimum weight maximum matching of *H* can be found in $O(p^3)$ time using the Hungarian algorithm. Finally, $MCPS _\varphi$ for the labeled circles in *G*(*A*, *B*) can be computed in *O*(*f*(*n*)) time. Combining these results we obtain an $O(p^{2}f(P)+p^3+f(n))$ time algorithm for computing $MCPS _\varphi(G(A,B),\lambda )$.

Now suppose that $f(r)=O(r^t)$ for some constant $t\ge 1$. Let $a_{1},\ldots ,a_{p}$ and $b_{1},\ldots ,b_{p}$ denote the number of edges in *AA* and *BB* paths with $\sum _{i=0}^{p}a_{i}=P_{A}$, $\sum _{j=0}^{p}b_{j}=P_{B}$ and $P=P_{A}+P_{B}$.

$MCPS _\varphi$ for a union of an *AA* path and a *BB* path having *a* and *b* edges respectively can be obtained by computing $MCPS _\varphi$ for at most two circles on $a+b$ vertices due to Theorem 2. This can be done in less than $c(a+b)^{t}$ steps for some constant *c* using the $O(r^t)$ time algorithm for computing $MCPS _\varphi$ for a circle. $MCPS _\varphi$ for every pair of *AA* and *BB* paths of $G(A,B)'$ can be computed in a number of steps bounded by:

$$\begin{aligned}&\sum _{i=0}^{p}\sum _{j=0}^{p}c(a_{i}+b_{j})^{t}=c\sum _{i=0}^{p}\sum _{j=0}^{p} \sum _{l=0}^{t}\left( {\begin{array}{c}t\\ l\end{array}}\right) a_{i}^{l}b_{j}^{t-l}=c\sum _{l=0}^{t}\left( {\begin{array}{c}t\\ l\end{array}}\right) \sum _{i=. For0}^{p}\sum _{j=0}^{p}a_{i}^{l}b_{j}^{t-l}\\&\quad =c\sum _{j=0}^{p}\sum _{i=0}^{p}b_{j}^{t}+c\sum _{i=0}^{p}\sum _{j=0}^{p}a_{i}^{t} +c\sum _{l=1}^{t-1}\left( {\begin{array}{c}t\\ l\end{array}}\right) \sum _{i=0}^{p}a_{i}^{l}\sum _{j=0}^{p}b_{j}^{t-l}\\&\quad =cp\sum _{j=0}^{p}b_{j}^{t}+cp\sum _{i=0}^{p}a_{i}^{t}+c\sum _{l=1}^{t-1} \left( {\begin{array}{c}t\\ l\end{array}}\right) \sum _{i=0}^{p}a_{i}^{l}\sum _{j=0}^{p}b_{j}^{t-l}\\&\quad \le cp(\sum _{j=0}^{p}b_{j})^{t}+cp(\sum _{i=0}^{p}a_{i})^{t}+c \sum _{l=1}^{t-1}\left( {\begin{array}{c}t\\ l\end{array}}\right) (\sum _{i=0}^{p}a_{i})^{l}(\sum _{j=0}^{p}b_{j})^{t-l}\\&\quad \le c(pP_{B}^{t}+p P_{A}^{t})+p c\sum _{l=1}^{t-1}\left( {\begin{array}{c}t\\ l\end{array}}\right) P_{B}^{t-l}P_{A}^{l}=cp(P_{B}+P_{A})^t=cpP^t \end{aligned}$$

Thus, the weighting of *H* can be performed in $O(pP^{t})$ time. This provides us with an $O(pP^t+p^3+n^t)$ time algorithm for computing $MCPS _\varphi(G(A,B),\lambda )$. $\square$

### Lemma 5

#### **Lemma**

*For a constant* $t\ge 2$ *and an* $O(r^t)$ *time* $\alpha$-*approximation algorithm for* $\varphi$-MCPS *on a labeled circle on* *r* *vertices, there exists an* $O(n^{t+1})$ *time* $\alpha$-*approximation algorithm for* $\varphi$-MCPS *on a labeled breakpoint graph*.

#### *Proof*

In Theorem 2, $MCPS _\varphi$ on a simple cycle is expressed as the minimum of the $MCPS _\varphi$ for a set of corresponding circles. In Theorem 1, $MCPS _\varphi$ on a graph is expressed as the minimum of the sums of the $MCPS _\varphi$ for the simple cycles. We prove an auxiliary proposition establishing the following:

1. An $\alpha$-approximation for $MCPS _\varphi$ on a simple cycle can be obtained by taking the minimum of the $\alpha$-approximations for the corresponding circles.
2. An $\alpha$-approximation for $MCPS _\varphi$ on a graph can be obtained by taking the minimum of the sums of the $\alpha$-approximations for $MCPS _\varphi$ on the simple cycles.

#### **Proposition**

Take $k\in \mathbb {N}$ and two sets of positive numbers $\{q_{1}^{*},\ldots , q_{k}^{*}\}$ and $\{q_{1},\ldots , q_{k}\}$ with $q_{i}\le \alpha q_{i}^{*}$ for every *i*. The following inequalities hold:

1. $$min\{q_{i}|i\in \{1,\ldots ,k\}\}\le \alpha \text {min} \{q_{i}^{*}|i\in \{1,\ldots ,k\}\}$$
2. $$\sum _{i=0}^{k} q_{i}\le \alpha \sum _{i=0}^{k} q_{i}^{*}$$

#### *Proof*

Take *u* and *v* such that $q_{u}^{*}=min\{q_{i}^{*}|i\in \{1,\ldots ,k\}\}$ and $q_{v}=min\{q_{i}|i\in \{1,\ldots ,k\}\}$. By construction $q_{v}\le q_{u}\le \alpha q_{u}^{*}$ which proves the first inequality. For the second inequality it suffice to observe that $\sum _{i=0}^{k} q_{i}\le \sum _{i=0}^{k} \alpha q_{i}^{*}=\alpha \sum _{i=0}^{k} q_{i}^{*}.$ $\square$

A simple cycle of a breakpoint graph has at most one vertex of degree 2. This means that it has at most two corresponding circles (see Theorem 6). Taking the minimum of the $\alpha$-approximations for $MCPS _\varphi$ on these circles provides us with an $\alpha$-approximation for the simple cycle due to Theorem 6 and the first part of the proposition above. This way we obtain an $\alpha$-approximation algorithm for $\varphi$-MCPS on a simple cycle of a breakpoint graph that runs in $O(r^t)$ time where *r* is the number of the vertices in the simple cycle.

We can reuse the structure of a bipartite graph *H* presented in “[Sec Sec13]” section with the weights of the edges now being the $\alpha$-approximations for the $MCPS _\varphi$ on the corresponding simple cycles. Following the same reasoning as in “[Sec Sec13]” section, we know that the minimum cost maximum matching of *H* leads to a MAECD of a breakpoint graph minimizing the sum of the $\alpha$-approximations for the $MCPS _\varphi$ on its simple cycles. Combining Theorem 1, both parts of the proposition above, and the proof of Lemma 4, we obtain an $O(n^{t+1})$ time $\alpha$-approximation algorithm for $\varphi$-MCPS on a breakpoint graph. $\square$

### Lemma 6

#### **Lemma**

*If* $\rho _{\mathcal {O}}$ *is a minimum 2-break-length* $\mathcal {O}$-*scenario for a labeled circle* $(O,\lambda )$, *then* $\mathcal {T} (\rho _{\mathcal {O}})$ *is a planar tree on* $(O,\lambda )$. *In addition to that, for a planar tree* $\mathcal {T}$ *on* $(O,\lambda )$ *there exists an* $\mathcal {O}$-*scenario* $\rho _{\mathcal {O}}$ *such that* $\mathcal {T} (\rho _{\mathcal {O}})=\mathcal {T}$.

#### *Proof*

We prove the first statement by induction. It is trivially true if *O* has 2 vertices. We suppose it to be true for all the circles having less than 2*l* vertices and prove it for a circle having 2*l* vertices. Fix a minimum 2-break-length scenario $\rho _{\mathcal {O}}$. Its length is $l-1$ due to Lemma 1. The first $\mathcal {O}$-break of $\rho _{\mathcal {O}}$ transforms $(O,\lambda )$ into two vertex disjoint labeled circles $(O_{1},\lambda _1)$ and $(O_{2},\lambda _2)$ both having less vertices than *O*. The rest of the scenario $\rho _{\mathcal {O}}$ can be partitioned into $\rho _{\mathcal {O}}^{1}$ acting on the edges of $O_{1}$ and $\rho _{\mathcal {O}}^{2}$ acting on the edges of $O_{2}$. As $\rho _{\mathcal {O}}$ is a minimum 2-break-length scenario, $\rho _{\mathcal {O}}^{1}$ and $\rho _{\mathcal {O}}^{2}$ must also be minimum 2-break-length scenarios. By the inductive hypothesis, $\mathcal {T} (\rho _{\mathcal {O}}^{1})$ and $\mathcal {T} (\rho _{\mathcal {O}}^{2})$ are planar trees on $(O_{1},\lambda _{1})$ and $(O_{2},\lambda _{2})$ respectively. $\mathcal {T} (\rho _{\mathcal {O}})$ can be easily obtained from $\mathcal {T} (\rho _{\mathcal {O}}^{1})$ and $\mathcal {T} (\rho _{\mathcal {O}}^{2})$ by taking the union of their edges and adding an edge corresponding to the first 2-break of $\rho _{\mathcal {O}}$. This way we obtain a planar tree $\mathcal {T} (\rho _{\mathcal {O}})$ on $(O,\lambda )$ proving the first statement of the lemma.

Now define the *distance* of an edge $\{x,y\}$ in $\mathcal {T}$ as the minimum number of vertices between *x* and *y* in the fixed circular embedding of $\mathcal {T}$. For example, in the rightmost tree on the top of Fig. 5 the distance of the edge $\{w,z\}$ is one, because *t* is in between *w* and *z*, while the distance of the edge $\{x,y\}$ is 0. An edge is said to be *short* if its distance is 0. We prove an auxiliary proposition.

#### **Proposition**

*A planar tree* $\mathcal {T}$ *on* $(O,\lambda )$ *has a short edge incident to a leaf.*

#### *Proof*

Choose a leaf *x* in $\mathcal {T}$ incident to an edge of the minimum distance *d*. If $d\ne 0$, then in between the leaf and the vertex that it is adjacent to, there are *d* other vertices. Since $\mathcal {T}$ is planar on $(O,\lambda )$, it is easy to see that there is at least one other leaf among these *d* vertices, which contradicts the minimality of *x*. $\square$

Now take a short edge $\{x,y\}$ incident to a leaf *x* in $\mathcal {T}$. Take the black edges $\{u,v\}$ and $\{q,s\}$ in $(O,\lambda )$ labeled with *x* and *y* respectively and separated by a gray edge $\{v,q\}$. Perform an $\mathcal {O}$-break $(\{v,u\},x),(\{q,s\},y)\rightarrow (\{v,q\},x),(\{u,s\},y)$. resulting in two labeled circles. One of them is a terminal graph having two edges $\{v,q\}$ with the black one labeled with *x*. Remove the edge $\{x,y\}$ from $\mathcal {T}$. This way we have reduced the size of the problem. The number of the vertices in the circle was reduced by two and the number of the edges in the tree was reduced by 1. We iterate this procedure to construct a required scenario. See the bottom part of Fig. 5 for an example. $\square$

### Lemma 7

#### **Lemma**

(Farnoud and Milenkovic in [19]) *A minimum cost planar tree on a circle can be found in* $O(r^4)$ *time, where* *r* *is the number of vertices of a tree*.

#### *Proof*

Farnoud and Milenkovic pose the problem of sorting permutations by cost-constrained mathematical transpositions (a sorting scenario is called a *decomposition*) [19]. They define a cost function on the set of transpositions and treat the problem, called MIN-COST-MLD, of finding a minimum cost decomposition among the minimum length transposition decompositions of a permutation. They reduce this problem to finding a minimum cost planar tree on a circle, and propose the following $O(r^4)$ time dynamic programming algorithm for a tree having *r* vertices.

Enumerate the vertices 1 to *r* while respecting their order on the circle. Define *cost*(*i*, *j*) as the minimum cost of a planar tree on the vertices $\{i,\ldots , j\}$ for $1\le i<j\le r$ and set $cost(i,i)=0$ for $1\le i\le r$.

Take a planar tree $\mathcal {T}$ on the vertices $\{1,\ldots , r\}$. If $deg(1)=1$ and 1 is on the edge $\{1,q\}$, then the cost of $\mathcal {T}$ is equal to $\Phi (1,q)$ plus the costs of the subgraphs of $\mathcal {T}$ induced by the vertices $\{2,\ldots ,q\}$ and $\{q+1,\ldots , r\}$. If $deg(1)>1$, then take $q=\text {max}(\{u|\{1,u\}\text { belongs to }\mathcal {T} \})$ and $s=\text {max}(\{u|$ there is a path in $\mathcal {T}$ joining 1 and *u* but not visiting $q\})$. The cost of $\mathcal {T}$ is equal to $\Phi (1,q)$ plus the costs of the subgraphs of $\mathcal {T}$ induced by the vertices $\{1,\ldots ,s\}$, $\{s+1,\ldots , q\}$ and $\{q,\ldots ,r\}$. This observation provides us with the following equality:

$$\begin{aligned} cost(i,j)=\text {max}(cost(i,s)+cost(s+1,q)+cost(q,j)+\Phi (i,q)|\text { }i\le s<q\le j) \end{aligned}$$

for $1\le i<j\le r$, that leads to an $O(r^4)$ time dynamic programming algorithm for finding *cost*(1, *r*). $\square$

## Data Availability

Not applicable.
